# 2-(4-Hy­droxy­phen­yl)-3-meth­oxy-4*H*-chromen-4-one

**DOI:** 10.1107/S1600536813010982

**Published:** 2013-05-15

**Authors:** Illia E. Serdiuk, Michał Wera, Alexander D. Roshal, Jerzy Błażejowski

**Affiliations:** a Institute of Chemistry, V.N. Karazin National University, Svobody 4, 61077 Kharkiv, Ukraine; b Faculty of Chemistry, University of Gdańsk, J. Sobieskiego 18, 80-952 Gdańsk, Poland

## Abstract

In the title compound, C_16_H_12_O_4_, the substituent benzene ring and meth­oxy group are twisted relative to the 4*H*-chromene skeleton by 24.1 (1) and 61.3 (1)°, respectively. In the crystal, mol­ecules are connected by classical O—H⋯O and weak C—H⋯O hydrogen bonds, forming chains parallel to [201]. The 4*H*-chromene ring systems of adjacent mol­ecules are either parallel or inclined at an angle of 28.9 (1)°.

## Related literature

For general features of flavones and flavonols (derivatives of 3-hy­droxy-2-phenyl-4*H*-chromen-4-one), see: Demchenko (2009 ▶); Ma *et al.* (2012 ▶). For related structures, see: Wera *et al.* (2011*a* ▶,*b* ▶). For inter­molecular inter­actions, see: Aakeröy *et al.* (1992 ▶); Etter *et al.* (1990 ▶); Novoa *et al.* (2006 ▶). For the synthesis, see: Bader *et al.* (2003 ▶); Wera *et al.* (2011*b* ▶).
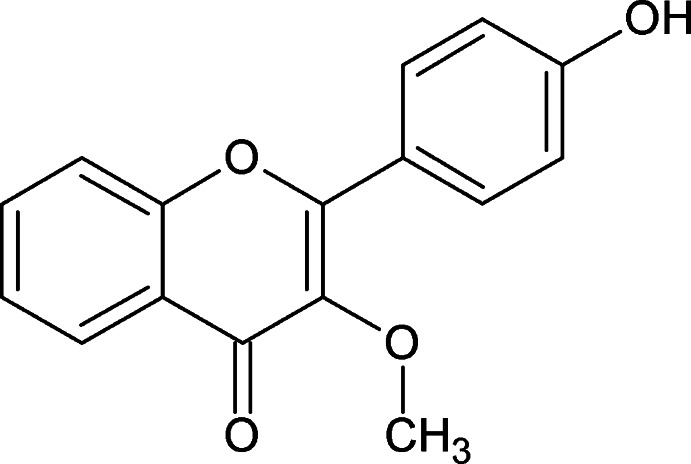


## Experimental

### 

#### Crystal data

C_16_H_12_O_4_
*M* *_r_* = 268.26
Monoclinic, 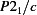

*a* = 8.7191 (5) Å
*b* = 8.8978 (4) Å
*c* = 16.706 (1) Å
β = 103.801 (6)°
*V* = 1258.65 (12) Å^3^
*Z* = 4
Mo *K*α radiation
μ = 0.10 mm^−1^
*T* = 295 K
0.6 × 0.45 × 0.25 mm

#### Data collection

Oxford Diffraction Gemini R Ultra Ruby CCD diffractometer
Absorption correction: multi-scan (*CrysAlis RED*; Oxford Diffraction, 2008 ▶) *T*_min_ = 0.943, *T*_max_ = 0.970
5100 measured reflections
2245 independent reflections
1801 reflections with *I* > 2σ(*I*)
*R*_int_ = 0.021

#### Refinement

*R*[*F* ^2^ > 2σ(*F* ^2^)] = 0.040
*wR*(*F* ^2^) = 0.107
*S* = 1.03
2245 reflections
185 parameters
H atoms treated by a mixture of independent and constrained refinement
Δρ_max_ = 0.14 e Å^−3^
Δρ_min_ = −0.18 e Å^−3^

### 

Data collection: *CrysAlis CCD* (Oxford Diffraction, 2008 ▶); cell refinement: *CrysAlis CCD*; data reduction: *CrysAlis RED* (Oxford Diffraction, 2008 ▶); program(s) used to solve structure: *SHELXS97* (Sheldrick, 2008 ▶); program(s) used to refine structure: *SHELXL97* (Sheldrick, 2008 ▶); molecular graphics: *ORTEP-3* for Windows (Farrugia, 2012) ▶; software used to prepare material for publication: *SHELXL97* and *PLATON* (Spek, 2009 ▶).

## Supplementary Material

Crystal structure: contains datablock(s) global, I. DOI: 10.1107/S1600536813010982/xu5696sup1.cif

Structure factors: contains datablock(s) I. DOI: 10.1107/S1600536813010982/xu5696Isup2.hkl

Supplementary material file. DOI: 10.1107/S1600536813010982/xu5696Isup3.cml

Additional supplementary materials:  crystallographic information; 3D view; checkCIF report

## Figures and Tables

**Table 1:** Hydrogen-bond geometry (Å, °)

*D*—H⋯*A*	*D*—H	H⋯*A*	*D*⋯*A*	*D*—H⋯*A*
O20—H20⋯O12^i^	0.93 (3)	1.77 (3)	2.648 (2)	157 (2)
C7—H7⋯O20^ii^	0.93	2.56	3.337 (3)	141

Symmetry codes: (i) 
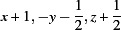
; (ii) 
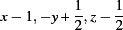
.

**Fig. 1.**
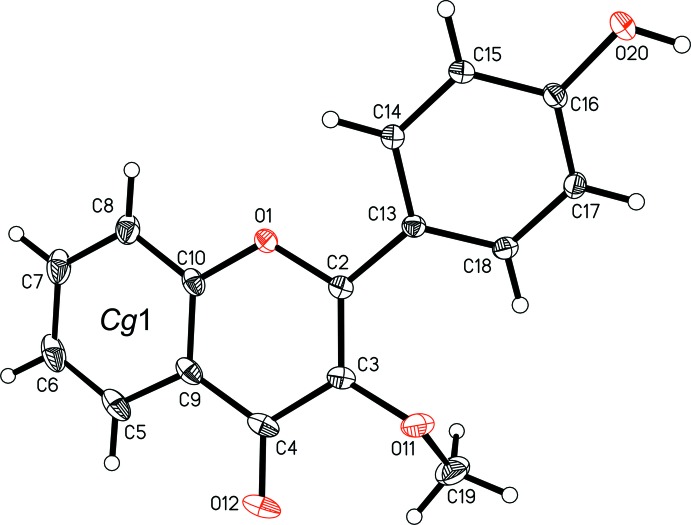
The molecular structure of the title compound showing the atom labeling scheme. Displacement ellipsoids are drawn at the 25% probability level, and H atoms are shown as small spheres of arbitrary radius.

**Fig. 2.**
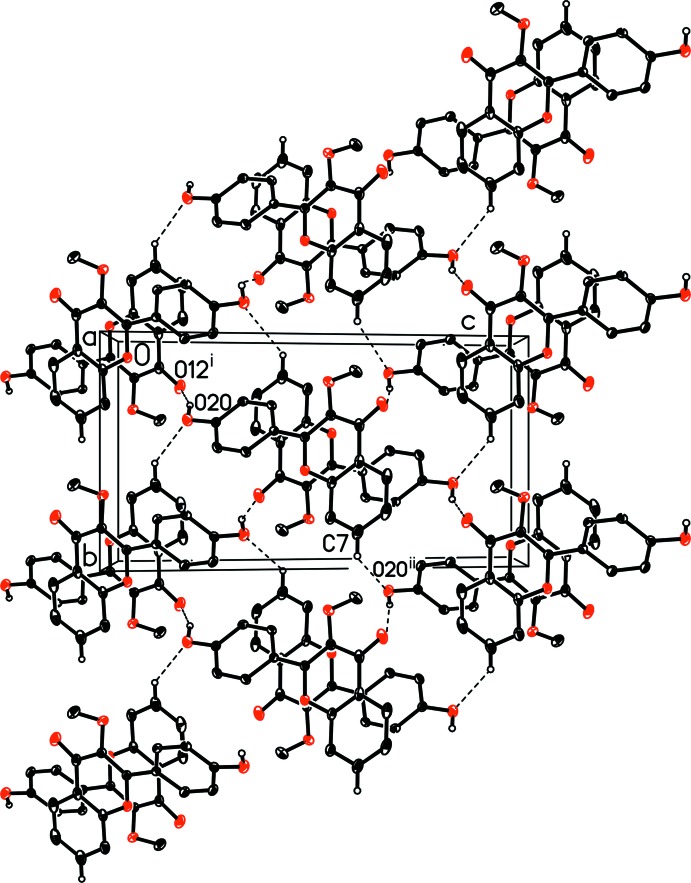
Molecular arrangement in the crystal structure. The O–H···O and C–H···O interactions are represented by dashed lines. H atoms not involved in interactions have been omitted. [Symmetry codes: (i) x + 1, –y – 1/2, z + 1/2; (ii) x – 1, –y + 1/2, z – 1/2.]

**Fig. 3.**
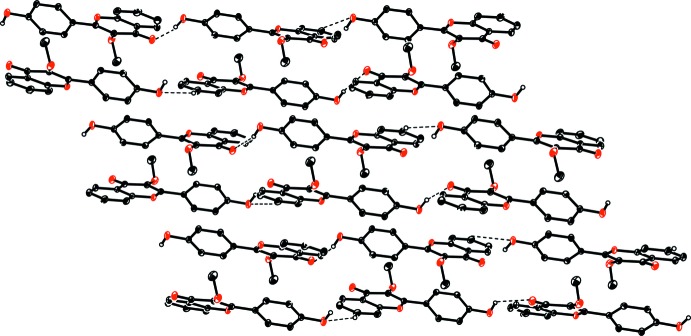
Layers in the crystal structure. The O–H···O and C–H···O interactions are represented by dashed lines. H atoms not involved in interactions have been omitted.

## supplementary crystallographic information

### Comment

Flavones (derivatives of 2-phenyl-4*H*-chromen-4-one) occur in numerous natural systems and have been thoroughly investigated because of their biological relevance (Ma *et al.*, 2012). Related to flavones, 3-hydroxy-2-phenyl-4*H*-chromen-4-ones (flavonols) exhibit dual fluorescence in condensed phases due to Excited State Intramolecular Proton Transfer (ESIPT) which makes the compounds interesting fluorescent sensors for analytical applications (Demchenko, 2009). Here we present the crystal structure of the flavonol derivative – 2-(4-hydroxyphenyl)-3-methoxy-4*H*-chromen-4-one – in which ESIPT does not occur. This makes the compound a convenient reference substance in investigations of emission phenomena in this group of compounds.

In the title compound (Fig.1), the bond lengths and angles characterizing the geometry of the 4*H*-chromen-4-one moiety are similar to those of other compounds of this group (Wera *et al.*, 2011*a*,*b*). With respective average deviations from planarity of 0.0309 (1) Å and 0.0037 (1) Å, the 4*H*-chromen-4-one and benzene ring systems are oriented at a dihedral angle of 24.1 (1)° [in the case of 3-hydroxy-2-(4-hydroxyphenyl)-4*H*-chromen-4-one this angle is 20.7 (1)° (Wera *et al.*, 2011*a*), and in 3-hydroxy-2-(4-methoxyphenyl)-4*H*-chromen-4-one it is 12.3 (1)° (Wera *et al.*, 2011*b*)]. The methoxy group is twisted relative to the 4*H*-chromen-4-one ring system through an angle of 61.3 (1)°.

In the crystal structure, molecules connected by a network of O–H···O (Aakeröy *et al.*, 1992), the C(10) motif (Etter *et al.*, 1990) and C–H···O (Novoa *et al.*, 2006) interactions (Table 1, Figs. 2 and 3), are arranged in layers (Fig. 2) that are dispersively stabilized in the crystal lattice (Fig. 3). The 4*H*-chromene cores are either parallel or oriented at an angle of 28.9 (1)°, while the benzene rings are parallel or inclined at an angle of 76.6 (1)°.

### Experimental

The title compound was synthesized in four steps. First, 1-(2-hydroxyphenyl)-3-{4-[(4-methoxybenzyl)oxy]phenyl}prop-2-en-1-one (chalcone) was prepared by the condensation (with the elimination of water) of 1-(2-hydroxyphenyl)ethanone with 4-[(4-methoxybenzyl)oxy]benzaldehyde in *N*-methylpyrrolidone/aqueous KOH (1/1 *v*/*v*), then precipitated by neutralizing the reaction mixture with aqueous HCl and recrystallized from methanol. Next, chalcone was oxidatively cyclized in a K_2_CO_3_/methanol/H_2_O_2_ mixture, yielding to 3-hydroxy-2-{4-[(4-methoxybenzyl)oxy]phenyl}-4*H*-chromen-4-one (Bader *et al.*, 2003; Wera *et al.*, 2011*b*). This product was then alkylated by dimethyl sulfate in an acetonitrile/K_2_CO_3_ mixture. Finally, the 3-methoxy-2-{4-[(4-methoxybenzyl)oxy]phenyl}-4*H*-chromen-4-one thus obtained was heated in acetic acid to give the title compound [2-(4-hydroxyphenyl)-3-methoxy-4*H*-chromen-4-one] with a yield of 34%. Pale yellow crystals suitable for X-ray investigations were grown from 1,4-dioxane solutions of the chromatographically purified (Silica Gel, 2-propanol/chloroform, 1/20 *v*/*v*) precipitate of the final product (m.p. = 506–507 K).

### Refinement

H atoms of C–H bonds were positioned geometrically, with C–H = 0.93 Å and 0.96 Å for the aromatic and methyl H atoms, respectively, and constrained to ride on their parent atoms with *U*_iso_(H) = *xU*_eq_(C) where *x* = 1.2 for the aromatic H and 1.5 for methyl H atoms. Hydroxy H atom was located on a difference Fourier map and refined isotropically with *U*_iso_(H) = 1.5*U*_eq_(O).

### Crystal data

**Table:**

C_16_H_12_O_4_	*F*(000) = 560
*M**_r_* = 268.26	*D*_x_ = 1.416 Mg m^−^^3^
Monoclinic, *P*2_1_/*c*	Mo *K*α radiation, λ = 0.7107 Å
Hall symbol: -P 2ybc	Cell parameters from 2245 reflections
*a* = 8.7191 (5) Å	θ = 3.3–25.1°
*b* = 8.8978 (4) Å	µ = 0.10 mm^−^^1^
*c* = 16.706 (1) Å	*T* = 295 K
β = 103.801 (6)°	Prism, pale yellow
*V* = 1258.65 (12) Å^3^	0.6 × 0.45 × 0.25 mm
*Z* = 4	

### Data collection

**Table:**

Oxford Diffraction Gemini R Ultra Ruby CCD diffractometer	2245 independent reflections
Radiation source: Enhance (Mo) X-ray Source	1801 reflections with *I* > 2σ(*I*)
Graphite monochromator	*R*_int_ = 0.021
Detector resolution: 10.4002 pixels mm^-1^	θ_max_ = 25.1°, θ_min_ = 3.3°
ω scans	*h* = −7→10
Absorption correction: multi-scan (*CrysAlis RED*; Oxford Diffraction, 2008)	*k* = −10→10
*T*_min_ = 0.943, *T*_max_ = 0.970	*l* = −18→19
5100 measured reflections	

### Refinement

**Table:**

Refinement on *F*^2^	Primary atom site location: structure-invariant direct methods
Least-squares matrix: full	Secondary atom site location: difference Fourier map
*R*[*F*^2^ > 2σ(*F*^2^)] = 0.040	Hydrogen site location: inferred from neighbouring sites
*wR*(*F*^2^) = 0.107	H atoms treated by a mixture of independent and constrained refinement
*S* = 1.03	*w* = 1/[σ^2^(*F*_o_^2^) + (0.0515*P*)^2^ + 0.1866*P*] where *P* = (*F*_o_^2^ + 2*F*_c_^2^)/3
2245 reflections	(Δ/σ)_max_ < 0.001
185 parameters	Δρ_max_ = 0.14 e Å^−^^3^
0 restraints	Δρ_min_ = −0.18 e Å^−^^3^

### Special details

**Table:**

Geometry. All e.s.d.'s (except the e.s.d. in the dihedral angle between two l.s. planes) are estimated using the full covariance matrix. The cell e.s.d.'s are taken into account individually in the estimation of e.s.d.'s in distances, angles and torsion angles; correlations between e.s.d.'s in cell parameters are only used when they are defined by crystal symmetry. An approximate (isotropic) treatment of cell e.s.d.'s is used for estimating e.s.d.'s involving l.s. planes.
Refinement. Refinement of *F*^2^ against ALL reflections. The weighted *R*-factor *wR* and goodness of fit *S* are based on *F*^2^, conventional *R*-factors *R* are based on *F*, with *F* set to zero for negative *F*^2^. The threshold expression of *F*^2^ > σ(*F*^2^) is used only for calculating *R*-factors(gt) *etc*. and is not relevant to the choice of reflections for refinement. *R*-factors based on *F*^2^ are statistically about twice as large as those based on *F*, and *R*- factors based on ALL data will be even larger.

### Fractional atomic coordinates and isotropic or equivalent isotropic displacement parameters (Å^2^)

**Table:**

	*x*	*y*	*z*	*U*_iso_*/*U*_eq_	
O1	0.26225 (13)	0.08174 (12)	0.03249 (6)	0.0445 (3)	
C2	0.31549 (18)	−0.06240 (17)	0.03302 (10)	0.0390 (4)	
C3	0.2506 (2)	−0.15818 (18)	−0.02953 (10)	0.0443 (4)	
C4	0.1209 (2)	−0.1136 (2)	−0.09642 (10)	0.0503 (5)	
C5	−0.0405 (2)	0.1077 (3)	−0.16006 (12)	0.0656 (6)	
H5	−0.0915	0.0491	−0.2046	0.079*	
C6	−0.0777 (3)	0.2560 (3)	−0.15735 (13)	0.0762 (7)	
H6	−0.1540	0.2979	−0.2001	0.091*	
C7	−0.0028 (3)	0.3448 (3)	−0.09160 (13)	0.0729 (6)	
H7	−0.0295	0.4459	−0.0906	0.087*	
C8	0.1101 (2)	0.2854 (2)	−0.02795 (11)	0.0589 (5)	
H8	0.1607	0.3449	0.0163	0.071*	
C9	0.07400 (19)	0.0433 (2)	−0.09613 (10)	0.0488 (5)	
C10	0.14684 (19)	0.1346 (2)	−0.03126 (10)	0.0451 (4)	
O11	0.29961 (17)	−0.30466 (13)	−0.02436 (8)	0.0603 (4)	
O12	0.05411 (16)	−0.20337 (16)	−0.15021 (8)	0.0712 (4)	
C13	0.43909 (17)	−0.09082 (16)	0.10765 (9)	0.0358 (4)	
C14	0.4500 (2)	0.00252 (18)	0.17590 (10)	0.0460 (4)	
H14	0.3772	0.0799	0.1733	0.055*	
C15	0.5654 (2)	−0.01740 (19)	0.24656 (10)	0.0483 (4)	
H15	0.5694	0.0456	0.2914	0.058*	
C16	0.67592 (19)	−0.12985 (17)	0.25180 (10)	0.0403 (4)	
C17	0.66761 (19)	−0.22373 (17)	0.18547 (10)	0.0417 (4)	
H17	0.7414	−0.3003	0.1885	0.050*	
C18	0.55044 (19)	−0.20481 (16)	0.11453 (10)	0.0406 (4)	
H18	0.5458	−0.2697	0.0704	0.049*	
C19	0.3675 (3)	−0.3531 (2)	−0.09013 (14)	0.0818 (7)	
H19A	0.2869	−0.3571	−0.1405	0.123*	
H19B	0.4130	−0.4511	−0.0778	0.123*	
H19C	0.4481	−0.2835	−0.0962	0.123*	
O20	0.78716 (16)	−0.14223 (15)	0.32342 (8)	0.0600 (4)	
H20	0.867 (3)	−0.209 (3)	0.3198 (15)	0.090*	

### Atomic displacement parameters (Å^2^)

**Table:**

	*U*^11^	*U*^22^	*U*^33^	*U*^12^	*U*^13^	*U*^23^
O1	0.0465 (7)	0.0475 (7)	0.0342 (6)	0.0080 (5)	−0.0007 (5)	−0.0006 (5)
C2	0.0394 (9)	0.0405 (9)	0.0364 (9)	−0.0031 (7)	0.0078 (7)	−0.0004 (7)
C3	0.0477 (10)	0.0457 (9)	0.0382 (10)	−0.0106 (8)	0.0074 (8)	−0.0029 (7)
C4	0.0425 (10)	0.0709 (12)	0.0363 (10)	−0.0205 (9)	0.0075 (8)	−0.0040 (8)
C5	0.0418 (10)	0.1093 (18)	0.0412 (11)	−0.0004 (11)	0.0011 (9)	0.0086 (11)
C6	0.0557 (13)	0.123 (2)	0.0467 (13)	0.0313 (13)	0.0047 (10)	0.0234 (13)
C7	0.0751 (14)	0.0945 (16)	0.0489 (13)	0.0405 (13)	0.0144 (11)	0.0157 (11)
C8	0.0638 (12)	0.0716 (13)	0.0405 (11)	0.0243 (10)	0.0109 (9)	0.0053 (9)
C9	0.0357 (9)	0.0756 (12)	0.0339 (9)	−0.0045 (8)	0.0062 (7)	0.0050 (8)
C10	0.0365 (9)	0.0655 (11)	0.0325 (9)	0.0077 (8)	0.0069 (7)	0.0073 (8)
O11	0.0815 (10)	0.0453 (7)	0.0497 (8)	−0.0114 (6)	0.0069 (7)	−0.0083 (6)
O12	0.0654 (9)	0.0919 (10)	0.0484 (8)	−0.0313 (8)	−0.0021 (7)	−0.0173 (7)
C13	0.0363 (8)	0.0346 (8)	0.0356 (9)	−0.0031 (7)	0.0068 (7)	−0.0008 (6)
C14	0.0451 (9)	0.0458 (9)	0.0427 (10)	0.0103 (8)	0.0018 (8)	−0.0065 (7)
C15	0.0497 (10)	0.0511 (10)	0.0389 (10)	0.0079 (8)	−0.0001 (8)	−0.0113 (8)
C16	0.0391 (9)	0.0412 (8)	0.0371 (9)	−0.0008 (7)	0.0020 (7)	0.0038 (7)
C17	0.0413 (9)	0.0362 (8)	0.0471 (10)	0.0060 (7)	0.0092 (8)	0.0029 (7)
C18	0.0458 (10)	0.0370 (8)	0.0387 (9)	−0.0021 (7)	0.0097 (8)	−0.0056 (7)
C19	0.1109 (19)	0.0646 (13)	0.0691 (15)	0.0072 (13)	0.0199 (14)	−0.0224 (11)
O20	0.0549 (8)	0.0682 (9)	0.0459 (8)	0.0154 (6)	−0.0100 (6)	−0.0035 (6)

### Geometric parameters (Å, º)

**Table:**

O1—C10	1.3627 (18)	C9—C10	1.382 (2)
O1—C2	1.3633 (19)	O11—C19	1.433 (3)
C2—C3	1.362 (2)	C13—C18	1.390 (2)
C2—C13	1.462 (2)	C13—C14	1.395 (2)
C3—O11	1.368 (2)	C14—C15	1.368 (2)
C3—C4	1.443 (2)	C14—H14	0.9300
C4—O12	1.239 (2)	C15—C16	1.377 (2)
C4—C9	1.455 (3)	C15—H15	0.9300
C5—C6	1.362 (3)	C16—O20	1.3530 (19)
C5—C9	1.399 (2)	C16—C17	1.376 (2)
C5—H5	0.9300	C17—C18	1.378 (2)
C6—C7	1.384 (3)	C17—H17	0.9300
C6—H6	0.9300	C18—H18	0.9300
C7—C8	1.371 (3)	C19—H19A	0.9600
C7—H7	0.9300	C19—H19B	0.9600
C8—C10	1.383 (3)	C19—H19C	0.9600
C8—H8	0.9300	O20—H20	0.93 (3)
			
C10—O1—C2	121.13 (13)	C9—C10—C8	122.28 (16)
C3—C2—O1	120.33 (14)	C3—O11—C19	114.71 (15)
C3—C2—C13	129.24 (15)	C18—C13—C14	117.18 (14)
O1—C2—C13	110.41 (12)	C18—C13—C2	123.70 (14)
C2—C3—O11	118.76 (15)	C14—C13—C2	119.10 (14)
C2—C3—C4	121.84 (16)	C15—C14—C13	121.37 (15)
O11—C3—C4	119.09 (14)	C15—C14—H14	119.3
O12—C4—C3	122.05 (18)	C13—C14—H14	119.3
O12—C4—C9	122.48 (17)	C14—C15—C16	120.58 (15)
C3—C4—C9	115.47 (15)	C14—C15—H15	119.7
C6—C5—C9	120.2 (2)	C16—C15—H15	119.7
C6—C5—H5	119.9	O20—C16—C17	123.49 (15)
C9—C5—H5	119.9	O20—C16—C15	117.29 (15)
C5—C6—C7	120.58 (19)	C17—C16—C15	119.22 (15)
C5—C6—H6	119.7	C16—C17—C18	120.29 (14)
C7—C6—H6	119.7	C16—C17—H17	119.9
C8—C7—C6	120.7 (2)	C18—C17—H17	119.9
C8—C7—H7	119.6	C17—C18—C13	121.35 (14)
C6—C7—H7	119.6	C17—C18—H18	119.3
C7—C8—C10	118.22 (19)	C13—C18—H18	119.3
C7—C8—H8	120.9	O11—C19—H19A	109.5
C10—C8—H8	120.9	O11—C19—H19B	109.5
C10—C9—C5	117.95 (18)	H19A—C19—H19B	109.5
C10—C9—C4	119.33 (15)	O11—C19—H19C	109.5
C5—C9—C4	122.71 (17)	H19A—C19—H19C	109.5
O1—C10—C9	121.69 (16)	H19B—C19—H19C	109.5
O1—C10—C8	116.01 (15)	C16—O20—H20	112.6 (15)
			
C10—O1—C2—C3	−1.9 (2)	C4—C9—C10—O1	0.3 (2)
C10—O1—C2—C13	179.79 (13)	C5—C9—C10—C8	0.0 (3)
O1—C2—C3—O11	−175.76 (14)	C4—C9—C10—C8	178.69 (17)
C13—C2—C3—O11	2.2 (3)	C7—C8—C10—O1	178.60 (17)
O1—C2—C3—C4	−2.3 (2)	C7—C8—C10—C9	0.1 (3)
C13—C2—C3—C4	175.66 (15)	C2—C3—O11—C19	−120.39 (19)
C2—C3—C4—O12	−174.71 (16)	C4—C3—O11—C19	65.9 (2)
O11—C3—C4—O12	−1.2 (3)	C3—C2—C13—C18	23.8 (3)
C2—C3—C4—C9	5.1 (2)	O1—C2—C13—C18	−158.16 (14)
O11—C3—C4—C9	178.60 (15)	C3—C2—C13—C14	−157.69 (17)
C9—C5—C6—C7	0.1 (3)	O1—C2—C13—C14	20.4 (2)
C5—C6—C7—C8	0.0 (3)	C18—C13—C14—C15	0.0 (2)
C6—C7—C8—C10	−0.1 (3)	C2—C13—C14—C15	−178.62 (15)
C6—C5—C9—C10	−0.1 (3)	C13—C14—C15—C16	0.8 (3)
C6—C5—C9—C4	−178.73 (19)	C14—C15—C16—O20	179.61 (16)
O12—C4—C9—C10	175.76 (16)	C14—C15—C16—C17	−0.9 (3)
C3—C4—C9—C10	−4.1 (2)	O20—C16—C17—C18	179.71 (15)
O12—C4—C9—C5	−5.6 (3)	C15—C16—C17—C18	0.3 (2)
C3—C4—C9—C5	174.56 (16)	C16—C17—C18—C13	0.5 (2)
C2—O1—C10—C9	2.9 (2)	C14—C13—C18—C17	−0.7 (2)
C2—O1—C10—C8	−175.60 (15)	C2—C13—C18—C17	177.91 (14)
C5—C9—C10—O1	−178.42 (15)		

### Hydrogen-bond geometry (Å, º)

**Table:**

*D*—H···*A*	*D*—H	H···*A*	*D*···*A*	*D*—H···*A*
O20—H20···O12^i^	0.93 (3)	1.77 (3)	2.648 (2)	157 (2)
C7—H7···O20^ii^	0.93	2.56	3.337 (3)	141

Symmetry codes: (i) *x*+1, −*y*−1/2, *z*+1/2; (ii) *x*−1, −*y*+1/2, *z*−1/2.
